# Exploring Pediatric Neuroblastoma: A Comprehensive Analysis of Adrenal and Nonadrenal Tumor Characteristics in the United States

**DOI:** 10.1055/s-0045-1807243

**Published:** 2025-04-02

**Authors:** Ghaith AlWawi, Mohammad Omar Alrefai, Mohd Zaki Al-Wawi, Asma Qasim, M. Bakri Hammami

**Affiliations:** 1 Department of Clinical Sciences, College of Medicine, University of Sharjah, Sharjah, United Arab Emirates; 2 Department of Nephrology, Mayo Clinic, Rochester, Minnesota, United States; 3 Department of Pediatrics, Shaikh Khalifa Medical City, Abu Dhabi, United Arab Emirates; 4 Department of Medicine, Cleveland Clinic Abu Dhabi, Abu Dhabi, United Arab Emirates; 5 Department of Hematology and Oncology, H. Lee Moffitt Cancer Center, Tampa, Florida, United States; 6 USF Health Morsani College of Medicine, Tampa, Florida, United States

**Keywords:** neuroblastoma, adrenal, nonadrenal, survival outcomes, prognostic factors, population-based study, pediatric oncology

## Abstract

**Background and Purpose:**

Neuroblastoma (NB) is an aggressive malignant tumor arising from a primitive neural crest origin. While the significance of tumor location in survival outcomes is recognized, it remains inadequately explored. This study provides a comprehensive analysis of the survival and characteristics of pediatric adrenal and nonadrenal NBs in the United States.

**Methods:**

A retrospective analysis of pediatric primary NB between 1975 and 2016 was conducted using the Surveillance, Epidemiology, and End Results (SEER) database. Univariate and multivariate regression analyses were used to determine prognostic variables.

**Results:**

A total of 4,554 patients were included, comprising 52% males ( *n* = 2,385) and 79.2% Caucasians ( *n* = 3,569). The median age of the patients was one year (range: 0–19 years). In all, 44.4% ( *n* = 1,996) of the patients had adrenal NB and 55.6% ( *n* = 2,496) patients had nonadrenal NB. Adrenal NB was significantly more prevalent among males and those presenting at a younger age ( *p* < 0.001). Adrenal NB was more likely to have a higher grade and distant metastasis at diagnosis ( *p* < 0.001). Nonadrenal NB, female sex, surgical resection, and later year of diagnosis were associated with improved survival ( *p* < 0.001).

**Conclusion:**

This study highlights important factors that are helpful for prognostication of NB patients in the United States. Tailored approaches considering tumor site are crucial for effective management of NB.

## Introduction

Neuroblastoma (NB) is a highly aggressive form of pediatric cancer originating from neural crest–derived cells. 1 2 Up to 90% of NB cases are diagnosed by the age of 5 years. It is notably responsible for 15% of all pediatric cancer-related fatalities in the first year of life alone. 1 NB may arise in any structure of neural crest origin, predominantly within the adrenal medulla or the paraspinal ganglia. 3 4 Other primary sites include the abdomen/retroperitoneum, neck, thorax, and pelvis. 5 NB is broadly categorized into adrenal and nonadrenal NBs due to the abundance of adrenal tumors. 1 NB exhibits a wide genetic diversity, 4 with tumors combining chromosomal gains or losses with candidate driver mutations. 6 7 8

The diverse primary tumor sites coupled with genetic variance contribute to a wide array of clinical manifestations and outcomes. These range from highly aggressive, treatment-resistant metastatic forms to more benign cases that in certain instances may undergo spontaneous regression. 9 Other factors such as staging, age at diagnosis, histological characteristics, tumor size, and genetic makeup are also influential in determining the clinical course and outcome of NB. 10 Consequently, attempts have been made to categorize NB cases into stratified risk groups to inform treatment protocols and prognostic estimations more effectively. 3 4 10 11 12 13

In the recent years, significant strides were made in our understanding of the molecular biology and pathogenesis of the tumor, allowing the development of more targeted treatment modalities and improving the survival of these patients significantly. 2 14 15 16 17 18 Small studies showed an association between the location of NB and survival. 19 20 This association remains poorly understood.

The primary aim of this study is to analyze the survival outcomes in pediatric patients with NB in the United States, with a specific focus on the impact of the primary tumor site—adrenal versus nonadrenal from 1975 up till 2016.

## Methodology

### Data Source

The Surveillance, Epidemiology, and End Results (SEER) database was used for this study. The SEER program is sponsored by the National Cancer Institute (NCI) and collects data on cancer incidence and mortality from 22 population-based registries covering approximately 48% of the U.S. population since 1973 ( http://seer.cancer.gov/seerstat ). Data were obtained from the SEER-18 registries (November 2020 submission) using SEER*stat software (8.3.8).

### Study Population

Data were examined from 1975 to 2016 to identify patients with primary NB. We included pediatric patients (0–20 years) with the International Classification of Disease for Oncology (3rd edition) codes: 9500: Neuroblastoma, NOS and 9490: Ganglioneuroblastoma. Year of diagnosis was specified from 1975 to 2016. Only first primary tumors were included. On initial screening, we identified 4,744 patients. We excluded cases with no histological confirmation, no active follow-up (death certificate only), alive with zero survival months, incomplete survival dates, and/or unknown cause of death. A total of 4,553 cases were included in the final study. No duplicate cases were found in our study population.

Outcome was categorized as “alive,” “dead attributable to this cancer diagnosis,” or “dead from other cause.” Race was categorized as white, black, or others including American Indian, Alaska Native, Asian, Pacific Islander, and unknown. Year of diagnosis of the tumor was split into two groups (those diagnosed before the year 2000 and those diagnosed in the year 2000 or later) to account for changes in treatment era. Other variables including sex, age at diagnosis, staging, and follow-up were also collected.

### Statistical Analysis

Frequency, survival data, and patient characteristics were obtained using SEER*Stat software (8.3.6) case listing session. Data were entered into IBM SPSS statistical package v.27 (SPSS Inc, Chicago, IL, United States). Descriptive statistics including mean, standard deviation, and percentages were used to detail patients' characteristics. Overall survival was calculated from the time of diagnosis to the time of event or last follow-up using Kaplan–Meier product limit curves and log-rank testing. The effects of continuous variables on survival were assessed using Cox proportional hazards regression. After univariable analysis was performed, factors with *p* -values of less than 0.05 were entered into a multivariable model. Proportional hazards assumption was evaluated by log-rank Kaplan–Meier plots. Hazard ratio (HR) and 95% confidence intervals (CIs) were reported for all significant factors in the multivariable model.

For further statistical analysis of the data, chi-squared test was used to compare categorical variables, whereas Fischer's exact test was used when the frequency was less than 5. *t* -test and analysis of variance (ANOVA) test were used as appropriate to compare continuous data. The Kruskal–Willis test was used instead of ANOVA when data were not normally distributed. All statistical tests were two sided. A *p* -value of less than 0.05 was considered statistically significant.

## Results

### Frequency Distributions by Primary Site

The distribution of various demographic and clinical variables was examined based on the primary site of NB. Table 1 summarizes these variables.

A significant difference was observed in gender distribution between adrenal and nonadrenal cases. Males comprised 55.7% of adrenal cases compared to 49.6% in nonadrenal cases ( *p* < 0.001). White patients were more likely to have nonadrenal NB compared to other races ( *p* = 0.006). A higher percentage of adrenal NB was diagnosed after the year 2000, compared to nonadrenal NB (71.3 vs. 62.1%; *p* < 0.001). Regarding disease stage, adrenal cases were more likely to present at a distant stage (73.1%) than nonadrenal cases (34.7%; *p* < 0.001). Males were more likely present at a distant stage than females ( *p* = 040). They were also more likely to present with undifferentiated (grade IV) tumor than females ( *p* = 0.003). While no significant difference was observed in the surgical intervention rates between the two groups ( *p* = 0.359), adrenal cases were significantly more likely to receive radiotherapy (31.5 vs. 19.7%; *p* < 0.001) and chemotherapy (76.3 vs. 58.5%; *p* < 0.001) compared to nonadrenal cases.

**Table 1:** Description of the cohort characteristics stratified by location

Variable		Primary site		*p* -value
		Adrenal	Nonadrenal	
Gender	Male	1,111 (55.7%)	1,238 (49.6%)	**<** **0.001**
	Female	885 (44.3%)	1,258 (50.4%)	
Race	Whites	1,536 (77.5%)	1,982 (80.5%)	**0.006**
	Blacks	258 (13%)	308 (12.5%)	
	Others a	189 (9.5%)	172 (7.0%)	
Stage	Local	32 (14.1%)	323 (30.9%)	**<** **0.001**
	Regional	29 (12.8%)	361 (34.5%)	
	Distant	166 (73.1%)	363 (34.7%)	
Grade	Well differentiated; grade I	33 (3.5%)	95 (9.4%)	**<** **0.001**
	Moderately differentiated; grade II	16 (1.7%)	30 (3%)	
	Poorly differentiated; grade III	668 (70.2%)	679 (67%)	
	Undifferentiated; grade IV	235 (24.7%)	210 (20.7%)	
Year of diagnosis	< 2000	573 (28.7%)	946 (37.9%)	**<** **0.001**
	> 2000	1,423 (71.3%)	1,550 (62.1%)	
Surgery	Surgery performed	1,497 (75.9%)	1,829 (74.7%)	0.359
	Surgery not performed	475 (24.1%)	619 (25.3%)	
Radiotherapy	Received	628 (31.5%)	492 (19.7%)	**<** **0.001**
	Not received/unknown	1,368 (68.5%)	2,004 (80.3%)	
Chemotherapy	Received	1,522 (76.3%)	1,459 (58.5%)	**<** **0.001**
	Not received/unknown	474 (23.7%)	1,037 (41.5%)	

Note: Bold font indicates statistically significant values ( *p* < 0.05).

a Others: Hispanics, pacific islanders, and Asians.

### Survival Analysis

Survival rates were generally higher for nonadrenal cases compared to adrenal NBs. Specifically, the 2- and 5-year survival rates were 87 and 80%, respectively, for nonadrenal cases compared to 77 and 65%, respectively, for adrenal cases. Table 2 summarizes the 2- and 5-year survival stratified by the NB site.

Univariate survival analysis revealed that nonadrenal site, female sex, white race, and cases diagnosed after 2000 were associated with improved survival in all cases, while those who received chemotherapy and radiotherapy had lower overall survival. When stratified by site, female sex was associated with improved survival in nonadrenal NBs but not in adrenal cases. Similarly, white race was associated with improved survival in nonadrenal NBs only ( Table 3 ).

Cox multivariate analysis revealed that adrenal origin was associated with worse survival (HR: 1.63; CI: 1.457–1.823). Surgical intervention and more recent year of diagnosis were associated with improved survival outcomes in all groups. Female sex was significantly associated with better survival in nonadrenal NBs only, while race did not emerge as a significant prognostic factor in any of the three groups ( Table 4 ).

**Table 2:** Two- and five-year survival stratified by tumor site

Variable		Interval	Cumulative % of survival at the end of interval	
			Adrenal	Nonadrenal
Primary site		2 y	77	87
		5 y	65	80
Year of diagnosis	< 2000	2 y	64	79
		5 y	53	72
	> 2000	2 y	83	91
		5 y	71	86
Race	White	2 y	78	87
		5 y	66	81
	Black	2 y	78	84
		5 y	64	75
	Other	2 y	72	84
		5 y	59	78
Chemotherapy	Administered	2 y	73	81
		5 y	58	71
	No/unknown	2 y	92	94
		5 y	90	93
Radiotherapy	Administered	2 y	72	75
		5 y	53	62
	No/unknown	2 y	80	89
		5 y	71	85
Surgery	Performed	2 y	82	92
		5 y	70	87
	Not performed	2 y	62	70
		5 y	51	62

**Table 3:** Univariate (Kaplan–Meier) survival analysis based on tumor site

Variable			Overall				Adrenal	Nonadrenal
			Mean survival (95% CI)	*p* -value	Mean survival (95% CI)	*p* -value	Mean survival (95% CI)	*p* -value
Primary site		Adrenal	292 (280–305)	**<** **0.001**				
		Nonadrenal	375 (366–385)					
Demographics	Sex	Male	334 (323–344)	**0.004**	286 (270–302)	0.416	368 (355–381)	**0.02**
		Female	350 (339–361)		297 (279–315)		383 (370–396)	
	Race	White	347 (339–356)	**<** **0.001**	296 (282–310)	0.089	382 (371–392)	**0.005**
		Black	309 (287–331)		283 (252–314)		332 (303–361)	
		Other	306 (277–335)		253 (216–291)		350 (309–391)	
Treatments	Surgical treatment	Performed	373 (365–381)	**<** **0.001**	316 (302–330)	**<** **0.001**	414 (405–424)	**<** **0.001**
		Not performed	250 (234–267)		221 (197–245)		272 (250–294)	
	Radiotherapy	Administered	261 (246–277)	**<** **0.001**	228 (205–251)	**<** **0.001**	286 (264–308)	**<** **0.001**
		No/unknown	370 (361–378)		319 (305–333)		401 (391–410)	
	Chemotherapy	Administered	290 (280–300)	**<** **0.001**	249 (235–263)	**<** **0.001**	323 (309–337)	**<** **0.001**
		No/unknown	439 (430–449)		414 (395–433)		448 (438–458)	
Year of diagnosis		< 2000	299 (287–311)	**<** **0.001**	237 (217–256)	**<** **0.001**	334 (320–349)	**<** **0.001**
		> 2000	156 (153–159)		141 (136–146)		171 (167–175)	

Abbreviation: CI, confidence interval.

Note: Bold font indicates statistically significant values ( *p* < 0.05).

**Table 4:** Cox multivariate regression analysis of factors associated with survival of patients with neuroblastoma

Variable			Overall		Adrenal		Nonadrenal	
			*p* -value	Hazard ratio (95% CI)	*p* -value	Hazard ratio (95% CI)	*p* -value	Hazard ratio (95% CI)
Primary site		Nonadrenal	**<** **0.001**	1.0 (ref)	–		–	
		Adrenal		1.63 (1.457–1.823)				
Demographics	Sex	Male	**0.049**	1.0 (ref)	0.535	1.0 (ref)	**0.028**	1.0 (ref)
		Female		0.895 (0.802–0.999)		0.955 (0.824–1.106)		0.831 (0.705–0.981)
	Race	White	0.094	1.0 (ref)	0.415	1.0 (ref)	0.188	1.0 (ref)
		Black	0.064	1.159 (0.992–1.355)	0.26	1.13 (0.913–1.399)	0.132	1.193 (0.948–1.5)
		Other	0.172	1.141 (0.944–1.379)	0.398	1.108 (0.874–1.405)	0.224	1.215 (0.888–1.663)
Treatments	Surgical treatment	No/unknown	**<** **0.001**	1.0 (ref)	**<** **0.001**	1.0 (ref)	**<** **0.001**	1.0 (ref)
		Performed		0.471 (0.42–0.529)		0.566 (0.482–0.664)		0.385 (0.324–0.457)
	Radiotherapy	No/unknown	**<** **0.001**	1.0 (ref)	**<** **0.001**	1.0 (ref)	**<** **0.001**	1.0 (ref)
		Administered		1.576 (1.403–1.77)		1.402 (1.198–1.64)		1.752 (1.471–2.089)
	Chemotherapy	No/unknown	**<** **0.001**	1.0 (ref)	**<** **0.001**	1.0 (ref)	**<** **0.001**	1.0 (ref)
		Administered		2.905 (2.444–3.453)		3.102 (2.383–4.04)		2.681 (2.129–3.376)
Year of diagnosis		< 2000	**<** **0.001**	1.0 (ref)	**<** **0.001**	1.0 (ref)	**<** **0.001**	1.0 (ref)
		> 2000		0.532 (0.476–0.595)		0.559 (0.480–0.650)		0.508 (0.428–0.603)

Abbreviation: CI, confidence interval.

Note: Bold font indicates statistically significant values ( *p* < 0.05).

## Discussion

In the present study, we conduct a comprehensive analysis of survival rates in adrenal and nonadrenal pediatric NBs in the United States. Among the most important findings were the higher survival rates in nonadrenal NBs, marked improvements in survival rates, and hazard reduction for diagnoses made post-2000 and cases undergoing surgery.

The presented study found that nonadrenal NB cases had significantly better survival rates compared to adrenal cases after adjusting for sex, race, year of diagnosis, and treatment modality. These findings are consistent with previous studies that have also reported better outcomes in nonadrenal NB cases. 5 20 The explanation for this disparity is that adrenal NB is often associated with negative prognostic markers such as metastasis at diagnosis, presence of MYC-N amplification, 20 21 1p loss, 11q loss, 17q gain, or DNA copy number alterations. 21 22 Adrenal cases predominantly manifested at a distant stage (73.1%), in contrast to nonadrenal cases (34.7%). Adrenal cases were also more likely to present with an undifferentiated grade. The significant divergence in tumor stage and grade suggests that adrenal NBs may inherently be more aggressive or have delayed clinical manifestations, leading to advanced-stage presentation, and necessitating more aggressive diagnostic and treatment methods. 21 It is worth noting that stage and grade analysis was limited due to the unavailability of related data in many cases.

Adrenal cases underwent radiotherapy and chemotherapy more frequently than nonadrenal cases (31.5 vs. 76.3% and 19.7 and 58.5%, respectively), consistent with previously reported studies. 23 Chemotherapy and radiotherapy were associated with lower survival rates as these treatments are reserved for intermediate- to high-risk NB cases. 24 Conversely, surgical intervention showed the best survival outcomes, likely due to the fact that it is recommended as the frontline treatment in low-risk NBs. 25

Both adrenal and nonadrenal NB cases exhibited an overall balanced sex distribution, although adrenal NB showed a slight predominance in males (55.7%). The analysis further showed that females have a higher mean survival time than males. When stratified by site, this difference was statistically significant in nonadrenal NB only. Overall, the influence of sex on NB survival is not clear. While some studies have found that sex is not a significant prognostic factor, 26 others reported that the association between sex and survival is mediated by the stage of disease. 27 In our study, males were more likely to present with adrenal NB, distant stage, and undifferentiated grade than females, potentially explaining the survival difference. Further analysis of prognostic markers and genetic mutations between males and females is warranted.

Similarly, race was associated with survival in nonadrenal NB only. White patients may have higher survival compared to other races, although this difference was not significant on multivariate analysis. A previous study found that black patients with NB have a higher prevalence of high-risk disease than white patients. 28 Another study suggested that the poorer outcomes might be due to higher incidence of high-risk NB among black patients. 29 According to a recent study, no association between race and survival was concluded. Nevertheless, the survival rates of patients treated during 2000 to 2004 and 2005 to 2015 showed improvement compared to those treated prior to 2000, suggesting reduction of the racial disparity in survival rates. 26 Around the year 2000, there were significant changes in the understanding and management of NB. 14 30 Some of the major changes that occurred around this time includes the development of more intensive, multimodality approaches to treat patients who are classified as high risk. 12 The discovery of genetic alterations that drive tumor growth resulted in the development of targeted therapies that exploit these alterations. 14 Further prognostic refinement might be attributed to the development of the International Neuroblastoma Risk Group Staging System (INRGSS), which was first proposed in 2009. 11 Multiple biologic and immunologic agents were developed over the recent years for treatment of NB. For example, dinutuximab and eflornithine were approved for high-risk NB by the Food and Drug Administration (FDA) in March 2015 31 and December 2023, 32 respectively. These multimodal approaches have significantly increased long-term survival, with recent studies reporting long-term survival up to 50% compared with 15% prior to their implementation. 33 34 35 36 37

While this study offers valuable insights, it is not without limitations. The database provides limited information on the status of radiotherapy and chemotherapy treatments, which consequently restricts the extent to which definitive conclusions can be drawn concerning these variables. Additionally, the SEER database lacks data on adjunctive therapies that may have been administered in conjunction with primary treatments, thereby potentially impacting the interpretation of treatment efficacy. The SEER database accounts for approximately 48% of cancer cases in the United States, which may limit the generalizability of our findings. Moreover, the absence of tumor grading and staging information for a significant proportion of cases limits the comprehensiveness of the study.

## Conclusion

In summary, this study establishes the primary tumor site as a significant predictor of survival outcomes in pediatric NB in the United States. We provide a stratified analysis of risk factors associated with adrenal versus nonadrenal NBs. Survival rates of patients with NB continues to improve over the past years owing to improved diagnostic and treatment modalities.
